# Osteo-Porosis

**Published:** 1894-01

**Authors:** G. J. Bowker

**Affiliations:** Groton, N. Y.


### OSTEO-POROSIS.

By G. J. Bowker, V.S.

In reading the discussions on osteo-porosis at the New York State Veterinary Medical Society, I noticed several of the veterinarians attribute it to lack of the salts of lime. It may be of interest to them to hear a little of my experience.

Last summer I was in North Dakota, near the Red River, which country is noted for its alkaline water. The water is so thoroughly impregnated with the salts of lime, that in the summer the shirts of the men working in the hot sun, drinking large quantities of water and perspiring freely, will be all white from the sediment left after the sweat has dried up. Wherever a little pool dries up the ground will be left white, as if salt had been sprinkled over it.

On one farm I saw three cases of osteo-porosis, all in yearlings that had run out all the time with practically no grain ; two of them were killed, the other was still alive when I left, but in such a bad condition that I don't think it could possibly have lived the summer out.

I advance no theory, only report those cases hoping they may be of some use to the veterinary profession *

Groton, N. Y.,

December 13th, 1893.

---

* In two enzootics with which I have had to deal, osteo-porosis developed in limestone countries where the water was excessively hard, and irritating to man unaccustomed to it. —R. S. H., Ed.

